# Influence of Chloroform on the Blood

**Published:** 1848

**Authors:** 


Article X.—*Influence of Chloroform on the Blood.*

M. Gruby, by experiments made on dogs, has arrived at the following results, manifested by the blood when chloroform is inhaled:

1. The arterial blood is more red—at least, as red—where chloroform has been inhaled, than (or as) where it has not.

3. The venous blood in an animal under the influence of chloroform, is more red than non-chloroformized arterial blood, and nearly as scarlet as such blood when penetrated by chloroform.

2. The venous blood becames of a clear red color under the use of chloroform, losing its usual reddish-black tint.

Hence it would appear that chloroform, far from rendering the hue of arterial blood venous, augments the intensity of its red color; and more than this, that it imparts the arterial color to venous blood.

In his experiments, M. Gruby was careful to use an instrument which allowed a due supply of atmospheric air to mix with the vapor of chloroform in inhalation; and to the omission of this precaution he would, in a great measure, attribute the different results which have been obtained by others.

In a note communicated to the Academy by Bouisson, of Montpellier, ether inhalation is looked upon as preferable to that of chloroform in surgery, where an operation is of long duration, since, the author believes, fatal or mischievous consequences are more apt to attend the prolonged administration of chloroform. But where, on the contrary, an operation is short, chloroform, by reason of its prompt action, deserves the preference.

In the discussion on this communication, however, M. Velpeau advanced it as his opinion, that, in the hands of a prudent surgeon, chloroform is to be always preferred.—*London Lancet.*

